# *Aspergillus fumigatus* antigen-reactive Th17 cells are enriched in bronchoalveolar lavage fluid in severe equine asthma

**DOI:** 10.3389/fimmu.2024.1367971

**Published:** 2024-08-20

**Authors:** Valentin F. Wjst, Sabrina Lübke, Bettina Wagner, Claudio Rhyner, Maria-Christin Jentsch, Corinna Arnold, Katharina L. Lohmann, Christiane L. Schnabel

**Affiliations:** ^1^ Institute of Immunology, Faculty of Veterinary Medicine, Leipzig University, Leipzig, Germany; ^2^ Centre for Proper Housing of Ruminants and Pigs, Federal Food Safety and Veterinary Office (FSVO), Ettenhausen, Switzerland; ^3^ Department of Population Medicine and Diagnostic Sciences, College of Veterinary Medicine, Cornell University, Ithaca, NY, United States; ^4^ Christine Kühne Center for Allergy, Research, and Education (CK-CARE), Davos, Switzerland; ^5^ Swiss Institute of Allergy and Asthma Research (SIAF), Davos, Switzerland; ^6^ Department for Horses, Faculty of Veterinary Medicine, Leipzig University, Leipzig, Germany

**Keywords:** RAO, heaves, T cells, type 3, mold, allergen, cytokine, chemokine

## Abstract

**Introduction:**

Equine asthma (EA) is a common disease of adult horses with chronic respiratory pathology and common neutrophilic airway inflammation. It presents with hyperreactivity to hay dust components such as molds, and underlying dysregulated T cell responses have been suggested. Thus far, T cells have been analysed in EA with conflicting results and the antigen reactivity of T cells has not been demonstrated. Serological and epidemiological data point to the relevance of *Aspergillus fumigatus* as an antigen source in EA. Here, we aimed to identify and characterise *Aspergillus* antigen-reactive T cells in EA.

**Methods:**

Cryopreserved bronchoalveolar lavage cells (BALC) and peripheral blood mononuclear cells (PBMC) from healthy horses (HE, n=9) and those with mild-moderate (MEA, n=3) or severe asthma (SEA, n=8) were stimulated *in vitro* with the recombinant *A. fumigatus* antigens Asp f 1, or Asp f 7 combined with Asp f 8, to assess antigen reactivity, and with phorbol-12-myristat-13-acetate and ionomycin (P/i) to assess overall T cell reactivity. Stimulated cells were analysed by flow cytometry for CD4, CD8, IL-17, IL-4, and IFN-γ. Cytokine expression in all lymphocytes, and in CD4^+^ or CD8^+^ T cells, was quantified and compared between the groups. In BAL fluid (BALF), soluble cytokines and chemokines were quantified by bead-based assays.

**Results:**

Antigen restimulation of BALC with Asp f 1 or Asp f 7/8 provoked higher frequencies of IL-17^+^ lymphocytes, CD4^+^IL-17^+^ Th17 cells, and CD4^+^IL-4^+^ Th2 cells in SEA than in HE, whereas MEA and HE were similar. Antigen stimulation of PBMC did not result in group differences. P/i stimulation of BALC resulted in increased IL-17^+^ lymphocyte and CD4^+^IL-17^+^ Th17 cell frequencies in MEA compared with HE but the limited number of horses with MEA must be considered. P/i-stimulated PBMC from MEA or SEA contained more IL-17^+^ lymphocytes compared with HE. Cytokines were hardly detected in BALF and similar between the groups but CCL2 and CCL5 concentrations were increased in BALF from SEA or MEA, respectively, compared with HE.

**Conclusion:**

Horses with SEA have increased *Aspergillus* antigen-reactive Th17 cells in their airways, emphasising local T cell responses to this mold, which were quantified in EA for the first time here.

## 1 Introduction

Equine asthma (EA) is a common non-infectious respiratory disease of horses affecting adult individuals with typical signs such as dyspnoea, cough, and exercise intolerance. EA is recognised as one of the leading causes of compromised athletic capacity, contributes substantially to horses’ welfare impairments, and has a major economic impact (1, 2). EA shares many characteristics with human asthma, including mucus hypersecretion, bronchial wall remodelling, and bronchospasm (1, 3). This also suggests EA as a non-rodent animal model with naturally occurring pathology for some subtypes of human asthma to study immunological pathways, such as the incompletely understood pathogenesis of neutrophilic asthma (3).

The diagnosis of EA and categorisation into phenotypes [mild to moderate equine asthma (MEA) and severe equine asthma (SEA)] is based on history, clinical presentation, and endoscopy. Through endoscopy, mucus scoring is routinely carried out and bronchoalveolar lavage (BAL) can be performed on standing sedated horses (1, 2). SEA is characterised by the presence of respiratory distress at rest based on impaired lung function. Additionally, SEA usually entails severe neutrophilic inflammation identified by BAL cytology. MEA is characterised by mild clinical signs, such as exercise intolerance and an occasional cough in the absence of lung function impairment or dyspnoea at rest. Airway cytology in MEA appears heterogeneous, and mixed, mastocytic, eosinophilic, or mild neutrophilic inflammation can be apparent in the BAL cytology of affected horses (1, 2).

For human asthma, the European Academy of Allergy and Clinical Immunology recently suggested applying the newest nomenclature of hypersensitivity reactions to group endotypes, i.e., the underlying pathologic mechanisms that lead to phenotypically observable asthma (4, 5). Asthma as a hypersensitivity can be broadly classified as T2 vs. non-T2 according to the dominance of type-2 cytokines [interleukin (IL)-4, IL-5, IL-13] or the lack of such, respectively. It is not critical for the T2 classification if the type-2 cytokines are mainly derived from T helper type 2 cells (Th2) or from other sources, such as innate lymphoid cells type 2 (ILC2) (4, 5). The type-2 cytokine milieu favours the class switching and B cell secretion of immunoglobulin (Ig)E. Therefore, T2 is often accompanied by allergic immediate reactions based on allergen-specific IgE (4, 5). In humans, T2 is the most common endotype and it usually encompasses eosinophilic inflammation, which can be detected phenotypically in sputum or BAL cytology (4, 5). By contrast, non-T2 asthma summarises all endotypes without increased type-2 cytokines. The best described ones are T1, based on type-1 cytokines such as IFN-γ secreted by Th1 cells and ILC1, or T3 with mediators like IL-17 from Th17 cells and ILC3, and IL-8 (CXCL8) induced by IL-17 (4). T3 asthma is usually associated with a severe phenotype and neutrophilic cytology in humans, and rodent models (5).

This nomenclature is applicable to other species, including horses as a potential model for non-T2 asthma (6). To use horses as an appropriate model, and to improve targeted diagnostic and therapeutic approaches to EA, the underlying pathological mechanisms require detailed identification and endotype definition. However, only phenotype definitions (MEA and SEA) have reached a consensus for EA so far (1, 3, 6). The pathology of EA is currently considered a multifactorial process, similar to human asthma. Its pathogenesis and the fundamental immune dysregulation in EA have not yet been completely defined. The underlying chronic inflammation of the lower respiratory tract in EA is provoked by environmental triggers, mainly inhaled hay dust components (1, 2, 6).

To date, conflicting data exist on EA pathophysiology and several hypotheses regarding EA pathogenesis have been suggested (2, 6) ranging from data supporting allergic responses and a T2 endotype (7–11) to a non-T2 endotype (12–14). In parts, conflicting data might result from differences in methodological approaches, and from the inclusion of different phenotypes, MEA and SEA, or the lack of their differentiation. Nevertheless, several analyses have compared healthy and asthmatic horses’ BAL lymphocytes in detail, pointing to the relevance of T cells, at least in SEA pathology. These identified increased rates of apoptosis of CD4^+^ and CD8^+^ T lymphocytes in the airways of horses with EA using flow cytometry (15), increased type-2 cytokine expressing lymphocytes through *in situ* hybridisation (10, 11), or the lack of patterns of polarised cytokine expression in sorted BAL T cells from SEA and healthy horses (16). Several studies have pointed to increased type-3 mediators, e.g. IL-17A and CXCL8 in SEA. These were found in mediastinal lymph nodes by immunohistochemistry and bulk ribonucleic acid (RNA) sequencing, in BAL cells (BALC) analysed by flow cytometry, or BALC submitted to single-cell (sc) RNA sequencing (12–14). The studies, which differentiated cellular sources of IL-17, indicated increased Th17 frequencies (13, 14), which were in line with a T3 endotype in SEA. Analyses in MEA have not covered T cell responses or did not find clear implications of these (13), and the relevance of T cells in the pathogenesis underlying the MEA phenotype is unclear (1, 6). Polarisations of circulating blood lymphocytes did not match those of local airway lymphocytes in EA (13, 16) and point to the importance of local T cells, which can be analysed in routinely acquired BALC from horses’ lungs.

Although T cells in EA have been studied using multiple approaches, it has not been identified to which antigens these T cells react. The importance of specific antigens for T cell responses in EA are accordingly unclear (6). Particularly, it has not been narrowed down whether single antigens stimulate excessive clonal T cell responses in EA or whether they represent increased T cell responses to *any* immunogenic antigens a predisposed horse’s airways are exposed to. The latter case of broad hyperreactivity would inform antigen-independent analyses and therapeutic approaches. The former case of specific antigens could inform targeted avoidance strategies, antigen-specific therapeutic approaches in equine medicine, and facilitate well-defined provocation modelling. Of note, in human T2 asthma, provoking allergens have been characterised by IgE serology, and an increase in allergen reactive T cell responses in asthma was subsequently demonstrated, which is paralleled in mouse models (17, 18). However, for non-T2 asthma, conclusions are deducted from mouse models using selected aero-antigens and adjuvants, and translation to human asthma is more insecure (18).

Deducting from epidemiological and serology analyses of EA, it has been indicated that single antigen sources provoke EA specifically (1, 2, 6). Molds, such as *Aspergillus* species, are common contaminants in hay, and horses are regularly exposed to respirable components of them (6). Antibody-focused analyses, mainly based on IgE binding, indicated *A. fumigatus* as a likely antigen source in EA, particularly its allergens Asp f 7 and Asp f 8 (7, 8, 19–22). These could contribute to B- and T cell responses in cognate recognition and accordingly be T cell antigens, but T cell antigen reactivity has not been analysed in EA. Ig binding differences between SEA and healthy horses was detected more often for Asp f 8 than for Asp f 7 (7, 8, 21). However, this difference has not informed a clear priority between these two (22), and immunogenicity as T cell antigens has not been investigated in horses before.

Furthermore, the major allergen Asp f 1 (23, 24) may be of interest for EA, as this allergen has been shown to exacerbate asthma in humans (25). Nonetheless, Asp f 1 has been indicated as potentially relevant to EA by a few reports of elevated IgE and IgG in SEA (22, 26, 27). These reports found antibodies against Asp f 1 in healthy and SEA horse samples, but with quantitative differences between the groups (22, 26, 27). Other studies of allergen screening by microarrays included Asp f 1 but did not find significantly increased IgE against it in EA compared with heathy horse samples (7–9). Accordingly, Asp f 1 represents an immunogenic fungal protein, which provokes adaptive immune responses in most horses and humans.

We hypothesised that T cells from asthmatic horses react to *Aspergillus* antigens *in vitro* and that T cell responses with increased Th17 reactivity indicate a T3 endotype in the airways of horses with EA compared with healthy horses. To test this, we took a flow cytometric analysis approach after restimulation applicable for the analysis of equine T cells (13, 28, 29). To the best of our knowledge, this study is the first report on fungal antigen-reactive T cells in horses and demonstrates that direct quantification of antigen-reactive T cells is suitable in this large animal model of non-T2 asthma. It furthermore indicates fungal allergens as T cell antigens in SEA, which has not been attempted before.

## 2 Materials and methods

### 2.1 Horse classification and evaluation

Samples from horses (n=12) presented for respiratory examination to the Department for Horses, Faculty of Veterinary Medicine, Leipzig University, were used after informed consent of their owners and after the use of aliquots for cytology and clinical pathology. Eight horses were examined and sampled in the same way under the animal experiment permission number TVV22/20, file number 25-5131/490/23 (Landesdirektion Sachsen, Germany).

The Horse Owner Assessed Respiratory Signs Index (HOARSI) was used to assess the history of the horses in a summary index (range 1–4) (30, 31), and treatment before presentation was recorded. Physical examination at rest and after re-breathing was performed by a trained veterinary specialist, and a clinical score, adapted from Ivester et al., was deducted as described previously (13, 32). Heparinised venous blood was taken and the horses were sedated. Afterwards, endoscopy was conducted using a flexible video endoscope (G28-250, 10.4 mm diameter, Storz, Tuttlingen, Germany) and mucus was scored as established by Gerber et al. (33). BAL fluid (BALF) was acquired under sedation and topical anaesthesia through the instillation of saline (60 ml/100 kg body mass, B. Braun, Melsungen, Germany), and aspiration as previously described (13). To evaluate airway cytology, an aliquot of BALF was processed by cytospinning (5 min centrifugation 113 × g, Shandon Cytospin centrifuge, Thermo Fisher Scientific, Waltham, MA, USA). Cytospins were stained with either Diff-Quick (RAL Diagnostics, Martillac, France) or Toluidine Blue (0.25 mg/ml Toluidine Blue O, Sigma-Aldrich, Merck KGaA, Darmstadt Germany), and cytology was assessed by differentiating a minimum of 500 nucleated cells as recommended (2).

Horse classification was based on the established criteria of HOARSI (2, 30, 31), clinical presentation (1, 2, 11, 13, 32), mucus score (33), and BAL cytology (1, 2). Horses were classified as healthy (HE) when they showed no history of signs of asthma, had no clinical signs of a respiratory disease, and had normal BAL cytology with ≦ 0.5% eosinophils, ≦ 5% MC, and ≦ 8% PMN (2). If one of those parameters was not met, but horses did not show respiratory distress at rest, they were classified as MEA, and horses with a history of clinical signs of asthma, clinical findings at presentation including respiratory distress at rest, and abnormal neutrophilic BAL cytology were classified as SEA (1, 2). Horses that had received previous treatment with corticosteroids or had signs of other systemic diseases (e.g. fever) were excluded from the study.

### 2.2 Sample processing

Blood in sodium heparin vacuum tubes (Becton Dickinson GmbH, Heidelberg, Germany) was used to isolate peripheral blood mononuclear cells (PBMC) via density gradient centrifugation (Pancoll 1077, Pan-Biotech, Aidenbach, Germany) as described previously (13). BALF was passed over a cell strainer (ClearLine100 µm, Kisker Biotech, Steinfurt, Germany) and subsequently centrifuged as described previously (13). Cell-free BALF was frozen at −80°C.

Bronchoalveolar lavage cells (BALC) or PBMC were resuspended in medium [Dulbecco’s modified Eagle medium (DMEM) low glucose (1 g/l), 1% (v/v) non-essential amino acids, 2 mM L-glutamine, 50 μg/ml gentamicin, 100 U/ml penicillin, 100 μg/ml streptomycin, and 50 μM 2-mercaptoethanol, all from Sigma-Aldrich, and 10% (v/v) heat-inactivated fetal calf serum from Lonza, Cologne, Germany], adjusted to 4 x 10^7^/ml and cryopreserved with freezing medium (final 15% dimethylsulfoxide and 20% FCS in DMEM) using Nalgene Mr. Frosty Freezing Containers (Thermo Fisher Scientific, Darmstadt, Germany) as established by Sage and co-workers (34).

Cells were thawed by a short incubation at 37°C, immediately transferred into 15 ml cold medium using a wide-mouth pipet and centrifuged for 5 min (200 × g) at 4°C. The supernatant was discarded and the cells were washed again (400 x g) and resuspended in medium according to cell counts using a trypan blue viability evaluation and a Neubauer counting chamber method. Aliquots of the processed cells were kept on ice for 6 h for control purposes (freeze-thaw, f-t) or used for stimulation *in vitro*.

### 2.3 BALC and PBMC stimulation *in vitro*

We used BALC and PBMC of 20 horses (9 healthy, 3 MEA, and 8 SEA) for *in vitro* stimulation according to the availability of sufficient cell numbers (**Table 1**). Per well, 2.5 × 10^6^ cells in 250 µl (1 × 10^7^ live cells/ml medium) were aliquoted into a sterile polystyrene 96-well flat-bottom tissue culture plate (Thermo Fisher Scientific) and incubated at 37°C at 5% CO_2_. Stimulations were conducted *in vitro* (**Table 1**): all cells were assessed after thawing (f-t) as negative controls, and medium-incubated cells served as baseline expression controls. Antigen restimulation was performed with 10 µg/ml recombinant Asp f 1 and a mixture of 10 µg/ml each of Asp f 7 and Asp f 8 (Asp f 7/8). Accidentally, for PBMC stimulation, only 2.4 µg/ml Asp f 8 was used in the mix.

**Table 1:** Cell stimulations with 24 h incubation *in vitro* (n per set-up).

BALC	Medium	P/i^a^	Asp f 1^b^	Asp f 7/8 ^b^	f-t ^c^
HE	9	9	9	9	9
MEA	3	3	3	3	3
SEA	5	5	5	4	5
PBMC	Medium	P/i^a^	Asp f 1 ^b^	Asp f 7/8 ^b^	f-t ^c^
HE	7	7	7	5	7
MEA	3	3	3	2	3
SEA	8	8	8	7	8

a P/i PMA and ionomycin were added after 18 h, for the last 6 h of the total 24 h incubation *in vitro*;

b *Aspergillus* antigens were recombinantly produced in *E. coli* and purified as described previously (23, 24);

c f-t cells were kept on ice for 6 h after thawing and analysed as controls without stimulation.

There were insufficient cell numbers of asthmatic horse BALC to use Asp f 7 and Asp f 8 separately. These were therefore combined to represent the two most described antigens in EA, whereas Asp f 1 represented a very likely immunogen to provoke adaptive immune responses in all individuals. Phorbol-12-myristat-13-acetate (PMA) and ionomycin stimulation (P/i) served as positive controls and were also used assess the overall cytokine expression capacities of T cells (13, 29). For P/i, 25 ng/ml PMA (Sigma-Aldrich) and 1 µM ionomycin (Sigma-Aldrich) were added for the last 6 h of the 24 h only. During the last 6 h of the 24 h stimulation, 10 µg/ml brefeldin A was added to all incubated cells (Sigma-Aldrich).

After incubation on ice (f-t) or stimulation *in vitro*, cells were transferred into polystyrene V-bottom plates (Boettger GmbH, Bodenmais, Germany), stained with viability dye eFluor™506 (eBioscience™, Thermo Fisher Scientific), fixed with paraformaldehyde, and stored at 4°C for a maximum of 3 days.

### 2.4 Staining for flow cytometry and analysis

As previously established for equine BALC and PBMC (13, 28), cells were stained for surface markers in PBS containing 3% (v/v) FCS (Lonza), 0.1% sodium azide (Roth, Karlsruhe, Germany), and 0.5% (w/v) saponin (Roth) for intracellular cytokine staining using equine-specific monoclonal antibodies (**Table 2**). Data of 300,000 events per sample were acquired with a BD LSR Fortessa™ Cell Analyzer (BD Biosciences, Ashland, OR, USA) equipped with FACS Diva ™ 8.1.2 software (BD) for the PBMC and version 6.1.3. for the BALC.

**Table 2:** Antibodies used for flow cytometry.

Equine target molecule	Antibody clone	Fluorochrome	Reference of equine-specific binding
CD4	HB61A	DyLight488 ^a^	(35)
CD8	CVS8	DyLight405 ^a^	(35)
IL-17	76	AlexaFluor647 ^a^	(29)
IL-4	13G7	AlexaFluor555 ^a^	(36)
IFN-γ	38-1	Biotin ^a^, followed by PE-Cy7 streptavidin (BioLegend)	(37)

a conjugated in-house using Sulfo-NHS esters (Thermo Fisher Scientific).

Analysis was performed with FlowJo™ v10.9.0 software (FlowJo, LLC, BD): First, singlets were gated by forward scatter (FSC) height and area characteristics. Then, viability dye positive cells were excluded, leaving live cells. Of those, lymphocytes were selected by FSC and side scatter (SSC) characteristics. Lymphocytes were analysed for CD4 and CD8 distribution by spider gates. For the analysis of cytokines, quadrant gates were set according to f-t intra-individual controls (without cytokine expression) vs. CD4 or CD8.

The percentages of IL-17A^+^, IFN-γ^+^, or IL-4^+^ of the lymphocytes in medium were subtracted from those after stimulation of the same cells, resulting in a net percentage. CD4^+^cytokine^+^ and CD8^+^cytokine^+^ lymphocytes were quantified relative to all CD4^+^ or CD8^+^ lymphocytes, respectively, and medium subtraction was applied to these to also yield a net percentage. For the approach here, 300,000 cells acquired for flow cytometry yielded at least 20,000 live lymphocytes and, therefore, frequencies as low as 0.1% still comprised 20 cells.

For a subset analysis of the viabilities in different cell populations, an additional gating strategy used a different hierarchy: singlets—lymphocytes—CD4 vs. CD8—live gating by viability dye vs. SSC on each subset.

### 2.5 BALF soluble mediator quantification

Soluble mediators were quantified in undiluted cell-free BALF by bead-based assays as described previously: IFN-γ, IL-4, IL-10, and IL-17A (38) in a multiplexed assay; IL-1β, TNF-α, CCL2, CCL3, CCL5, and CCL11 in another multiplexed assay (39, 40); and soluble CD14 (sCD14) in a singleplex assay (41). All assays were performed at the Animal Health Diagnostic Center, College of Veterinary Medicine, Cornell University, Ithaca, NY, USA. Concentrations of IFN-γ and IL-17A were quantified in U/ml, and all other soluble mediators are reported in pg/ml.

### 2.6 Statistical analysis

The flow cytometric data were mainly not normally distributed, and non-parametric Mann–Whitney tests were applied to compare MEA vs. HE and SEA vs. HE without correction for multiple comparisons using GraphPad PRISM v9 software (GraphPad Software, La Jolla, CA, USA). The mediator concentrations in BALF were log-normally distributed according to Shapiro–Wilk tests and therefore log-transformed. Then, ANOVA was applied on the log-transformed data and groups were compared using Fisher’s least significant difference (LSD) tests (GraphPad PRISM). A p-value ≤0.05 was considered statistically significant.

## 3 Results

### 3.1 T cells in cryopreserved equine BALC

**Figure 1:**
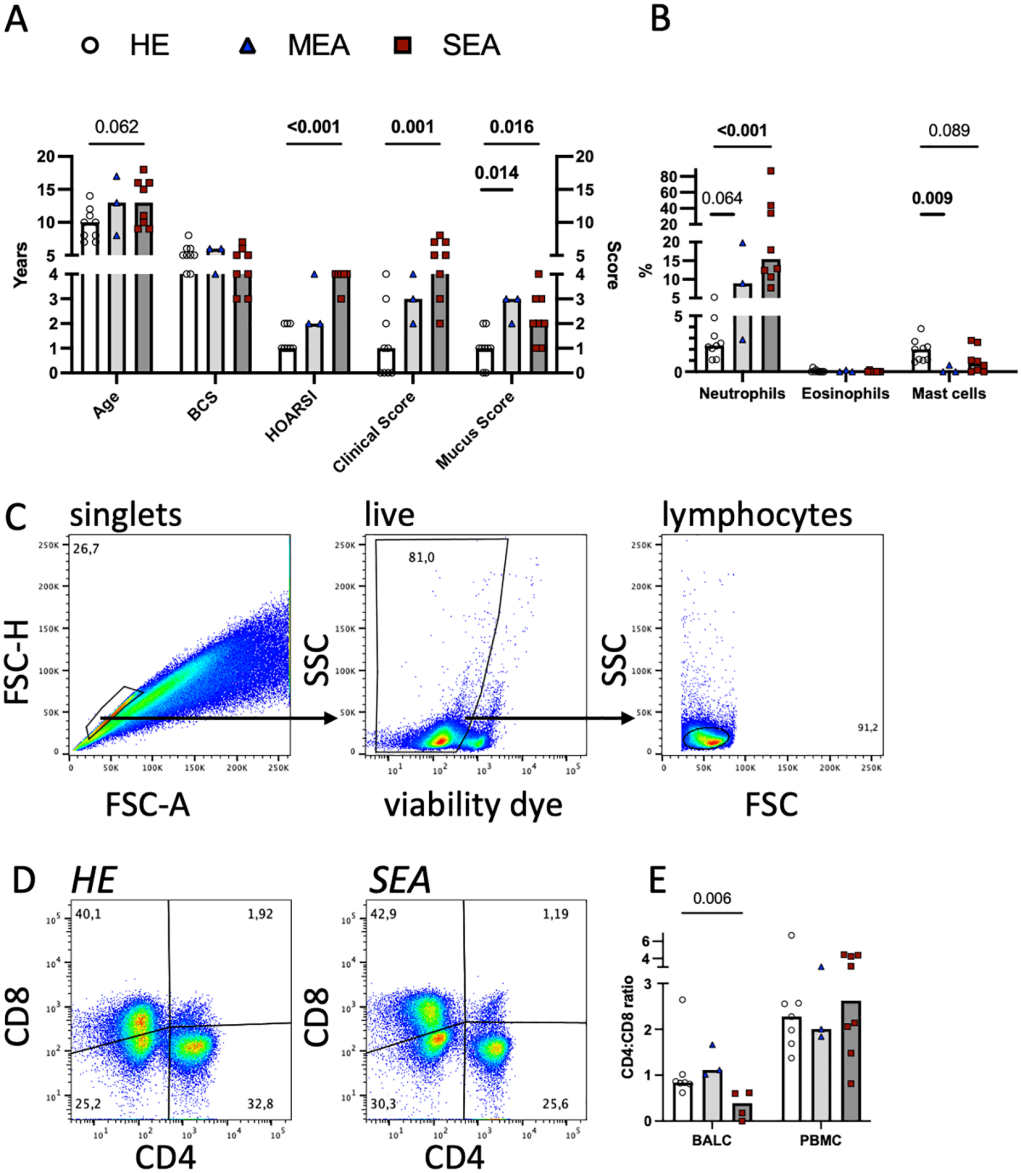
Flow cytometric T cell analyses in cryopreserved equine bronchoalveolar lavage cells. Horses were characterised as HE (n=9) or having MEA (n=3) or SEA (n=8). **(A)** The horses’ age, body condition score (BCS, range 1–9), history (HOARSI, range 1–4), clinical score (sum, range 0–21), and endoscopic mucus score (range 0–5) were assessed and are plotted. The bars represent group medians. Statistical comparisons using Mann–Whitney tests are indicated by lines with p-values if p<0.1. **(B)** BALC were microscopically differentiated on cytospins, and the percentages of the neutrophils, eosinophils, and mast cells of all leukocytes are plotted. Most asthmatic horses (MEA and SEA) had neutrophilic BAL cytology. **(C)** BALC and PBMC of the horses were cryopreserved, thawed, and analysed by flow cytometry. Singlets (conservative), live cells, and lymphocytes were gated in a hierarchical manner, as illustrated in a representative example BALC (HE, freeze-thaw). All further analyses were performed on lymphocytes. **(D)** CD4 and CD8 positive T cell proportions were determined by spider gates, as illustrated in representative examples of lymphocytes in BALC from HE and SEA. **(E)** The CD4:CD8 ratio was calculated for each sample (after thawing), except those from two horses (one HE and one SEA), the cells of which did not stain for CD8. SEA BALC had a lower CD4:CD8 ratio than HE BALC.

Twenty horses were thoroughly characterised as HE or having MEA or SEA according to their history, clinical presentation, and BAL cytology (**Figures 1A, B**). Horses with SEA tended to be older (median 13 years) than healthy horses (median 10 years). Most horses with asthma (MEA and SEA) had neutrophilic inflammation evident in BAL cytology (**Figure 1B**). Cryopreserved, thawed BALC and PBMC had median viabilities of 69% and 63%, respectively, as determined by flow cytometric viability dye analysis (**Figure 1C**). The viability of thawed BALC was similar between the groups but the viability of the PBMC from SEA exceeded that of HE (median 73% vs. 57%, p=0.006, **Supplementary Figure 1**). The live lymphocyte counts over all groups and samples analysed had medians of 20,000 (BALC) and 77,000 (PBMC) after 24 h incubation.

To evaluate the T lymphocytes in more detail, they were assessed for CD4 and CD8 expression. Two horses (one healthy and one SEA) did not have CD8 recognised by the established antibody used [clone CVS8 (10, 20, 27)], and for these, only CD4^+^ Th cells could be analysed separately.

The CD4:CD8 ratio in BALC lymphocytes was decreased in SEA compared with HE (**Figures 1D, E**, two horses without CD8 staining were excluded). The difference in CD4 and CD8 proportions was not reflected in the viabilities of the T cell subsets in BALC (**Supplementary Figure 1**). The increased PBMC viability of SEA was reflected in several subsets of PBMC lymphocytes but the difference was only statistically significant for CD4^-^CD8^-^ cells (**Supplementary Figure 1**, two horses without CD8 staining were excluded from the subset analysis).

### 3.2 *Aspergillus* antigens stimulate Th17 cells in BALC from severely asthmatic horses

**Figure 2:**
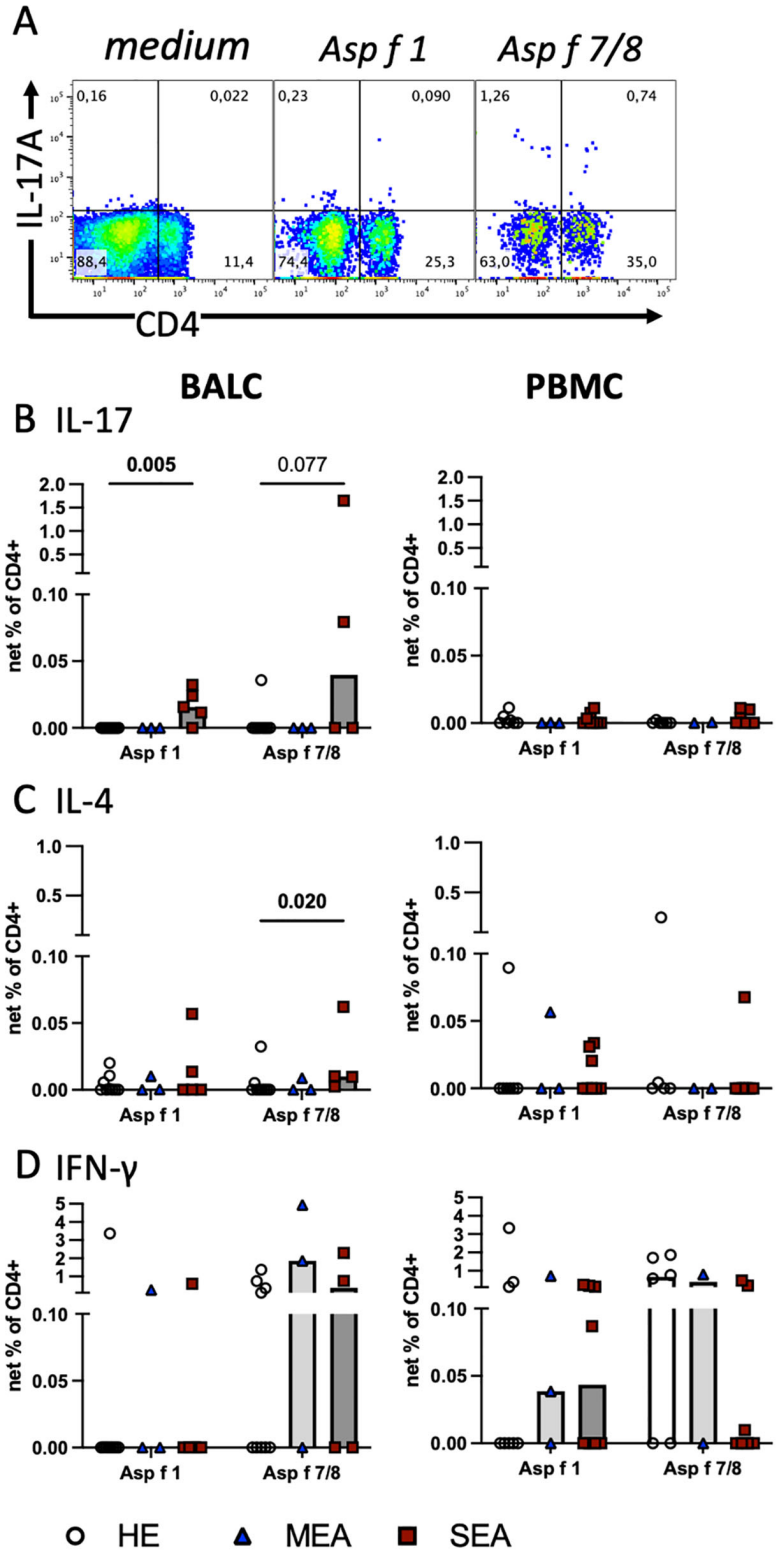
*Aspergillus* antigens stimulate Th cells *in vitro* and indicate increased local Th17 cells in SEA. Cryopreserved BALC and PBMC from HE (BALC, n=9; PBMC, n=7), MEA (n=3), or SEA (BALC n=5, PBMC n=8) were restimulated *in vitro* for 24 h with recombinant *A. fumigatus* antigens (Asp f 1, or mixed Asp f 7 and Asp f 8) and evaluated for cytokine expression by flow cytometry in comparison with medium-incubated controls (net percentage). **(A)** An example of SEA BALC gated on singlet live lymphocytes is shown (pseudocolour with enlarged dots for visibility). **(B–D)** Each cytokine was evaluated in CD4^+^ Th cells within BALC and PBMC. **(B)** Asp f 1 stimulated higher percentages of CD4^+^IL-17^+^ Th17 cells in SEA compared with HE BALC. Asp f 7/8 stimulation yielded a similar trend. **(C)** Asp f 7/8 stimulated more CD4^+^IL-4^+^ Th2 cells in SEA compared with HE BALC but **(D)** CD4^+^IFN-γ^+^ Th1 stimulations were comparable between the groups. The stimulation of PBMC did not yield group differences in Th responses to the antigens.

To assess antigen-reactive T cells, BALC or PBMC were restimulated *in vitro* with the recombinant allergens Asp f 1, or Asp f 7 combined with Asp f 8, for 24 h, and cytokine expression in T cells was quantified by flow cytometry in CD4^+^ (**Figure 2A**) and CD8^+^ lymphocytes (**Supplementary Figure 2**). The net percentages of the CD4^+^IL-17^+^, CD4^+^IL-4^+^, and CD4^+^IFN-γ^+^ cell subsets were compared and interpreted as antigen-reactive Th17, Th2, and Th1 cells, respectively. CD4^+^IL-17^+^ cell subsets were significantly increased after Asp f 1 stimulation (p=0.005, **Figure 2B**) and tended to be increased after Asp f 7/8 stimulation (p=0.077). This matched the increased net percentage of IL-17^+^ lymphocytes in Asp f 7/8-stimulated BALC (p=0.027, **Supplementary Figure 2C**). In contrast to BALC, IL-17 responses after antigen stimulation of PBMC were overall low and similar between the groups (**Figure 2B**). CD4^+^IL−4^+^ cell subsets were very few in BALC but they were increased (p=0.02) after Asp f 7/8 stimulation in BALC from SEA compared with HE (**Figure 2C**). IL-4 expression in PBMC (**Figure 2C**) was similar between the groups, except for increased CD8^+^IL-4^+^ cell subsets after Asp f 1 stimulation of PBMCs from SEA compared with HE (p=0.026, **Supplementary Figure 2**). IFN-γ expression in BALC (**Figure 2D**) and CD4^+^IFN-γ^+^ or CD8^+^IFN-γ^+^ cell subsets in PBMC were similar between the groups. However, IFN-γ^+^ lymphocyte net percentages in PBMC after Asp f 7/8 stimulation were decreased in SEA compared with HE (p=0.03, **Supplementary Figure 2E**).

**Figure 3:**
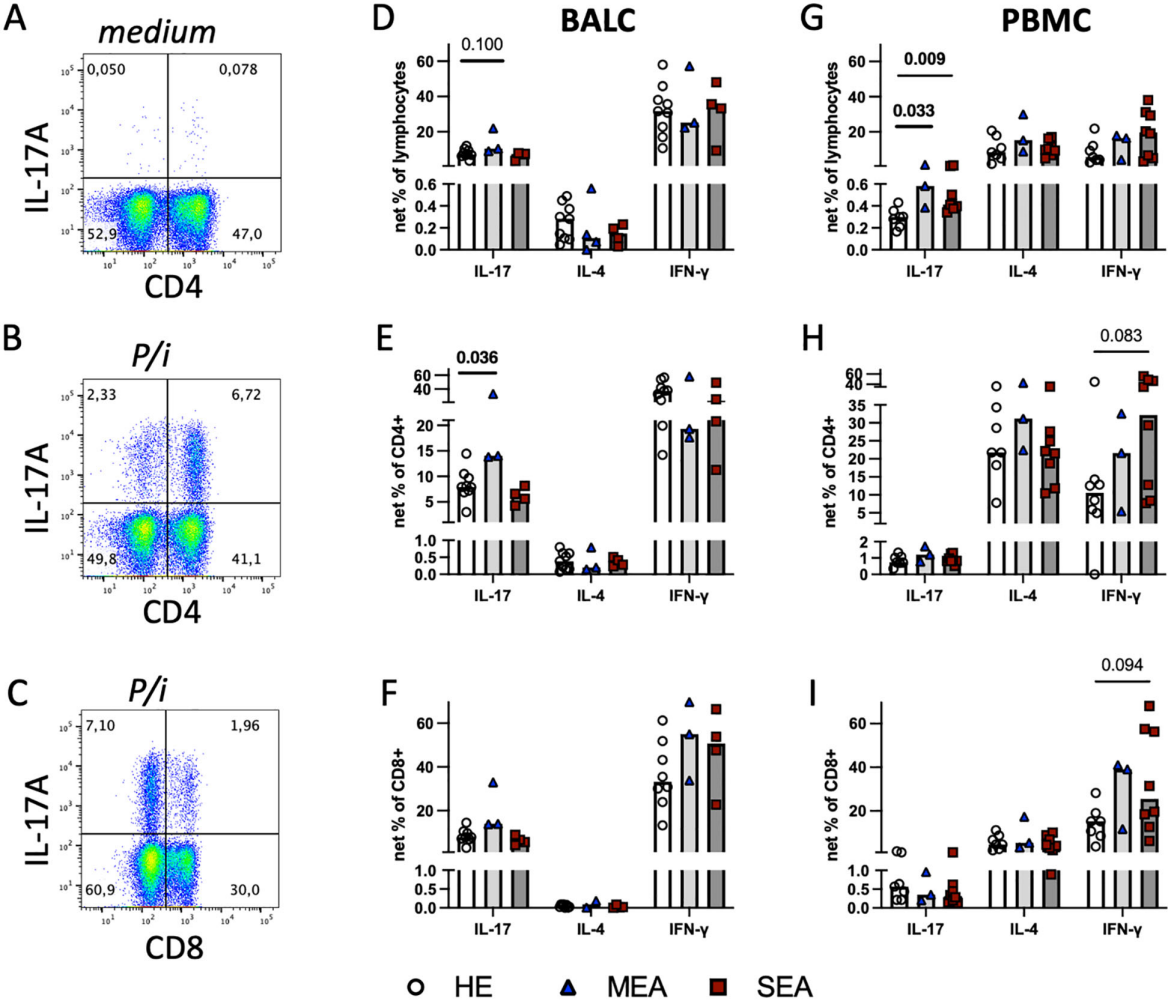
Polyclonal stimulation *in vitro* suggests local Th17 cell bias in MEA and indicates a systemic IL−17 increase in PBMC from MEA and SEA. BALC and PBMC from HE (BALC, n=9; PBMC n=7) or horses with MEA (n=3) or SEA (BALC n=5; PBMC n=8) were incubated *in vitro* for 24 h, with PMA and ionomycin added for the last 6 h (P/i) in comparison with medium alone, and analysed by flow cytometry. Representative examples of BALC (MEA) are shown. **(A)** Singlet-live-lymphocytes were gated and cytokine expression evaluated in quadrant gates, as illustrated for IL-17. P/i-stimulated expression was medium subtracted (net percentage) and analysed (e.g., CD4^-^IL-17^+^ + CD4^+^IL-17^+^) in BALC or PBMC lymphocytes **(D, G)**. **(B)** CD4^+^cytokine^+^ cell subsets were quantified in relation to all CD4^+^ lymphocytes [e.g., CD4^+^IL-17^+^/(CD4^+^IL17^+^ + CD4^+^IL-17^-^)] and medium subtracted [**(E)** BALC; **(H)** PBMC]. **(C)** CD8^+^cytokine^+^ lymphocyte subsets were analysed in the same manner as CD4^+^ and compared between the groups [**(F)** BALC; **(I)** PBMC]. **(D–I)** Bars represent group medians. Statistical comparisons in Mann-Whitney-tests are indicated by lines with p-values if p≦0.1. Group comparisons indicated increased CD4^+^IL-17^+^ net percentages in BALC from MEA compared with HE **(E)**. Net percentages of IL-17^+^ lymphocytes were increased in MEA or SEA PBMC compared with HE **(G)**.

The *A. fumigatus* antigens tested stimulated cytokine expression in CD4^+^ T cells in BALC and PBMC with the highest percentages of IFN-γ expression, followed by IL-17 in BALC or IL-4 in PBMC. Yet, group differences were most pronounced in CD4^+^IL-17^+^ antigen-reactive T cells, thereby agreeing with the general cytokine production after P/i stimulation (see **Figure 3**).

*Aspergillus* antigen-reactive Th17 cells were enriched in BALC from SEA compared with HE.

### 3.3 Polyclonal stimulation *in vitro* induces increased IL-17 expression in EA

To characterise the capacity of all T cells, BALC or PBMC were stimulated with PMA and ionomycin for the last 6 h of a 24 h incubation *in vitro* (P/i), and cytokine expression was quantified by flow cytometry (**Figures 3A, B**) in all lymphocytes (**Figures 3D, G**) within CD4^+^ (**Figures 3B, E, H**), or CD8^+^ T cells (**Figures 3C, F, I**). Of the three cytokines quantified, IFN-γ was expressed by most lymphocytes, followed by IL-17 in BALC or IL-4 in PBMC in all lymphocytes and T cell subsets analysed, but statistically significant group differences were only detected for IL-17 expression (**Figure 3**). In comparison with the increased antigen-reactive cells in SEA BALC, polyclonal stimulation pointed to increased Th17 responses with elevated CD4^+^IL-17^+^ cell subsets in MEA BALC, and the sample size in the MEA group warrants reserved interpretation. Yet, in PBMC, increased IL-17-expressing lymphocytes (**Figure 3G**) were observed in both MEA and SEA compared with HE but were not matched by the CD4^+^IL-17^+^ cell subsets, which did not have such group differences (**Figure 3H**).

All P/i stimulated BALC lymphocytes’ cytokine expressions were similar between the groups (**Figure 3D**) but CD4^+^IL-17^+^ cell subsets within the CD4^+^ BALC were also increased in MEA compared with HE (p=0.036), and the CD4^+^IL-4^+^ and CD4^+^IFN-γ^+^ cell subsets were similar between the groups (**Figures 3E, F**). There were fewer CD8^+^-cytokine-expressing lymphocytes than CD4^+^ (**Figures 3B, C**) but the CD8^+^IL-17^+^ and CD8^+^IFN-γ^+^ cell subsets were regularly detected, with similar percentages in all groups (**Figure 3F**). In PBMC, IL-17^+^ lymphocytes were increased in MEA and SEA compared with HE (p<0.05), whereas IL−4^+^ and IFN-γ^+^ were similar between the groups (**Figure 3G**). The percentages of IFN-γ^+^ of CD4^+^ and IFN-γ^+^ of CD8^+^ cell subsets in PBMC tended to be increased in SEA compared with HE (**Figures 3H, I**, p<0.1) but the relative expressions of IL-17 and IL-4 in the PBMC CD4^+^ and CD8^+^ T cell subsets were similar between the groups.

### 3.4 Soluble mediators in BALF

**Figure 4:**
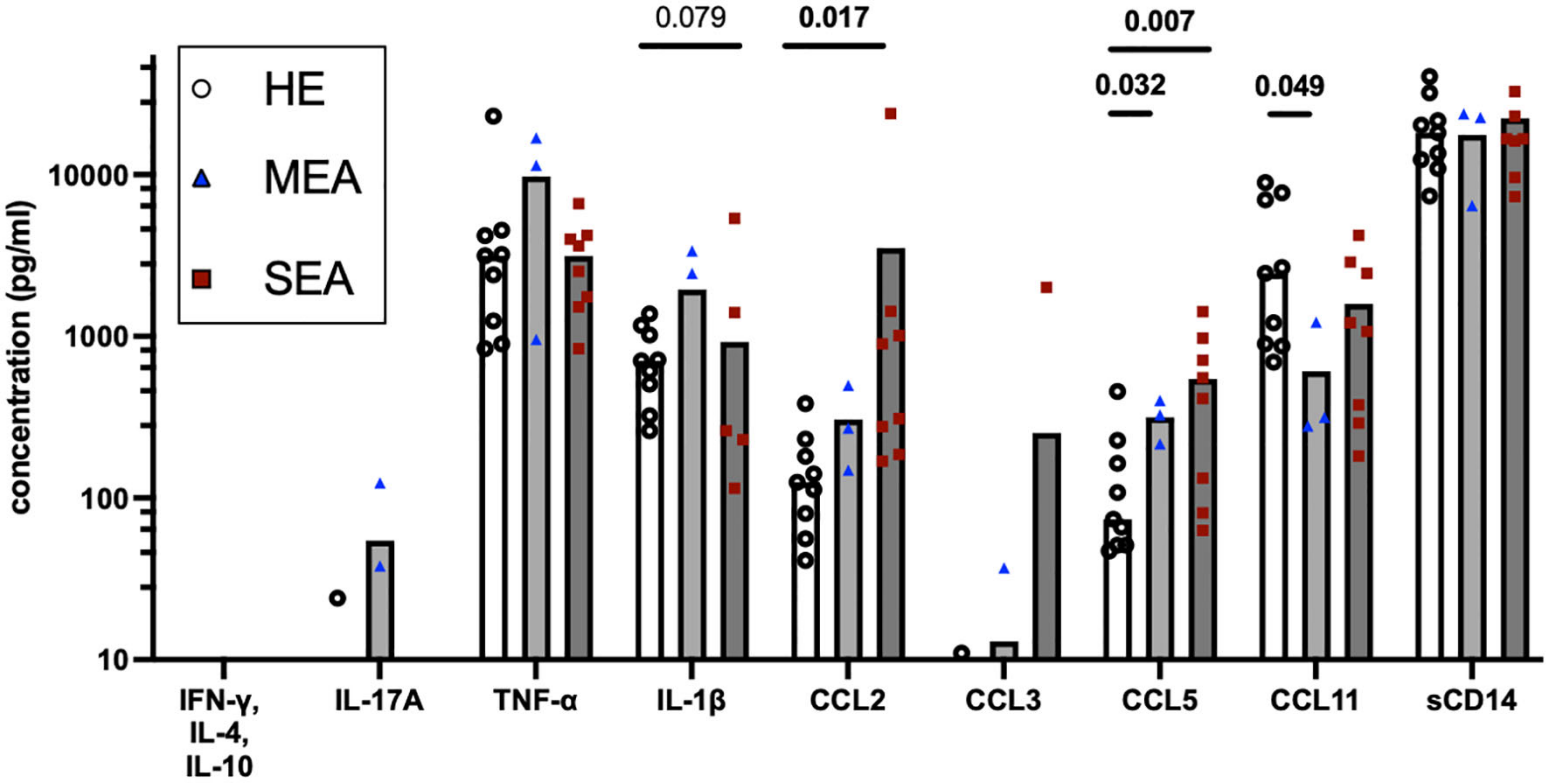
BALF cytokines and CCL chemokines in EA. Soluble cytokines, chemokines, and soluble (s) CD14 concentrations were quantified in BALF by bead-based assays and group comparisons (HE, n=9; MEA, n=3; SEA, n=8) analysed by ANOVA after log-transformation of the concentrations. IFN-γ, IL-4, and IL-10 were not detected and IL-17A was only detected in single BALF samples. CCL2 concentrations were increased in SEA. CCL5 concentrations were increased in MEA or SEA compared with HE. All other mediators quantified in BALF were not statistically significantly different between the groups.

To assess whether the IL-17 signature was reflected in secreted mediators in BALF as a different diagnostic option, these were quantified by bead-based assays (**Figure 4**). IFN-γ, IL-4, and IL-10 were not detected, and IL-17 was only detected in one BALF sample from HE and two BALF samples from MEA. Inflammatory cytokines, chemokines, and sCD14 were analysed in addition. The concentrations of TNF-α (median 4.8 ng/ml), IL-1β (median 0.9 ng/ml), and sCD14 (median 19.6 ng/ml) were easily detectable but similar between the groups. Chemokine concentrations differed between the different groups’ BALF. The CCL2 concentrations were increased in BALF from SEA (p=0.017) compared with HE, whereas CCL3 was only detected in a few samples from each group. CCL5 concentrations were increased in MEA and SEA compared with BALF from healthy horses (p<0.05) but CCL11 concentrations were decreased in MEA (p=0.049) (**Figure 4**). The same patterns were confirmed in a larger collection of BALF samples, with increased CCL5 in MEA but decreased CCL11 in SEA, and mostly undetectable T cell cytokines as the main findings (**Supplementary Figure 3**).

## 4 Discussion

Although dysregulated T cell responses have long been discussed as an important underlying mechanism (1, 6), antigen-reactive T cells have not been determined previously in EA. Here, we established *in vitro* restimulation of equine BALC and PBMC with recombinant *A. fumigatus* antigens followed by flow cytometric analysis of cytokine responses to evaluate equine T cell reactivity to fungal antigens. To our knowledge, this is the first report of the antigen reactivity of T cells in EA. This study can contribute to the assessment of the impact of specific T cell reactivity in EA as a potential model of T3 asthma.

Antibodies against *A. fumigatus* are elevated in SEA (7, 21, 22, 26, 27). Thus, antigens of *A. fumigatus* seem to be relevant in EA, and the three antigens tested here stimulated T cells from SEA-affected horses *in vitro*, confirming our hypothesis of T cell responses to specific fungal antigens in SEA. Asp f 1 is a major allergen in human allergic broncho-pulmonary aspergillosis and has been implied as an antigen but considered of minor relevance in EA according to serology (24–27). In the present study, it stimulated the most consistent elevation in Th17 responses of SEA BALC. The combination of Asp f 7 and Asp f 8, which have been more frequently reported as allergens in EA (7–9, 19, 21), resulted in a similar Th17 trend and also low Th2 responses *in vitro*, which were increased in SEA. If sufficient BALC are available, it will nevertheless be interesting to analyse Asp f 7 and Asp f 8 separately and identify each antigen’s relevance for Th reactivity in EA.

Despite the overall higher Th1 reactivities to the antigens compared with Th17 or Th2, the Th1 frequencies in BALC were similar between the groups in contrast to our previous report of concurrently increased Th17 and Th1 cells in SEA in a different cohort of horses (13). Throughout different studies with different cohorts of horses and methods, enriched local Th17 responses appear a most consistent finding in SEA and, therefore, T3 is the most supported endotype (12–14).

Antigen-reactive T cells were also detected in PBMC but these circulating T cells did not differentiate asthmatic from healthy horses, which points to a major relevance of local T cells in the airways in the pathologic mechanisms underlying EA.

Here, in samples from three horses with MEA, Asp f 1- or Asp f 7/8-reactive T cells were not increased compared with HE, leaving the question as to whether other T cell antigens are important in MEA in contrast to the repeatedly conveyed relevance of molds like *Aspergillus* spp. in SEA (1, 6–8, 19–21). Nevertheless, polyclonal T cell activation supported a possible role of local Th17 cells also in MEA in our current analysis, which has not been indicated for MEA before. This novel finding warrants further analyses of this phenotype considering the limited number of horses with MEA included for direct comparison here.

Remarkably, and consistent with our previous report (13) and the findings by Sage and co-workers using single-cell (sc) RNA sequencing (14), CD4^+^ Th17 cells were the main but not the only source of IL-17 in BALC, which was also expressed in CD8^+^ lymphocytes and CD4^-^CD8^-^ lymphocytes, particularly after P/i stimulation. Here, CD4^+^IL-17^+^ Th17 cells in BALC were the preferred cell phenotype, which differentiated EA from HE with statistical significance, whereas the overall IL-17 expressing lymphocytes or IL-17^+^CD8^+^ lymphocytes only reflected this after Asp f 7/8 stimulation. Still, IL-17 derived from CD8^+^ or CD4^-^CD8^-^ lymphocytes could contribute to the overall type-3 inflammatory response in EA, as supported by sc RNA sequencing of SEA BALC, which demonstrated increased IL17 expression in several T cell clusters, including those annotated as regulatory T cells and γδ T cells (14).

*In vitro* restimulation of equine PBMC was approached by others to study equine *Culicoides* hypersensitivity using recombinant *Culicoides* allergens and analysis of the secreted cytokines in cell culture supernatants (42). In that assay, bacterially expressed allergens induced background cytokine secretion, probably due to residual bacterial components and the non-specific stimulation of different cell types within PBMC, which likely diminished the analysis. However, differences between allergic and non-allergic horses’ PBMC were observed after insect cell or barley expressed allergens were used for stimulation (42). Non-specific stimulation did not seem to interfere in our present study using bacterially expressed recombinant *A. fumigatus* antigens, probably because the adhesion of macrophages *in vitro* and flow cytometry with conservative gating facilitated a lymphocyte-focused analysis here. This enabled the evaluation of different T cell subtype responses after stimulation with bacterially expressed recombinant antigens in the present study. Different antigen sources could additionally be considered for future studies.

Antigen reactive T cells and those stimulated by P/i indicated a Th17 bias in EA. However, antigen-reactive T cell responses in BALC were not merely reflective of the general proportion of Th17 cells, which could be stimulated with P/i. The latter were increased in MEA but not in SEA in the present cohort of horses within 24 h of incubation *in vitro* with 6 h of P/i stimulation. Th17 frequencies were also clearly increased in SEA compared with HE in our previous study with 3 h of P/i stimulation of fresh BALC (13). An increase of IL-17 by antigen-reactive and overall stimulated T cells in BALC from SEA or MEA, respectively, compared with HE was the most consistent pattern in this present study. This matched the neutrophilic inflammation observed in BAL cytology and previous descriptions of local IL-17 increases in SEA by others and us (12–14).

Other studies on mechanisms underlying EA analysed cytokine RNA expression (6, 10, 11, 43, 44) or secreted cytokines in BALF (38) to deduct T cell polarisations. In our direct comparison with flow cytometric analyses here, soluble T cell-associated cytokines were hardly detected in BALF despite the use of sensitive bead-based assays with a lower limit of detection in the pg/ml range (38, 40). The secreted cytokines in BALF represent the composition of mediators secreted by all cells in the lower airways, including myeloid cells and epithelial cells, which have a major impact. T cells are not the most numerous cell type in the airways and therefore Th signature cytokines are likely not secreted into the lumen in high amounts and be detectable in lavage fluid, which is dilute. BALF was considered potentially reflective of baseline expression but baseline expression of cytokines in medium-incubated BALC was low and similar between the groups here (data not shown), in contrast to the short-term stimulation with antigens or P/i *in vitro*. In summary of these considerations, T cell responses cannot be deducted from BALF cytokine concentration analyses alone or validly correlated.

However, inflammatory cytokines and chemokines were detected in BALF in the present study despite the dilute nature of this sample. Unexpectedly, inflammatory cytokines such as TNF-α and IL−1β, or the inflammatory marker sCD14 (41), were not increased in MEA or SEA BALF here, in contrast to previous reports on SEA (41, 43, 45).

Nevertheless, this is the first comprehensive analysis of CCL chemokine proteins in EA, and these chemokine concentrations differed between BALF from asthmatic horses and HE with increased CCL2 in SEA and increased CCL5 but decreased CCL11 in MEA and SEA. A CCL2 increase may point to the involvement of macrophage activation in EA. CCL2 is a neutrophil attractant and may stimulate neutrophils and Th17 cells, which express its receptor CCR2 in mice and humans (46, 47). Increased CCL5 in MEA and SEA corroborates T cell trafficking and supports T cell activation and involvement in both phenotypes of EA, as CCL5 is expressed by CD4^+^ Th cells and CD8^+^ T cells and regulated upon activation (39). Nonetheless, *CCL5* was also found as a differentially expressed gene in BALC T cells analysed by sc RNA sequencing in a comparison between HE and SEA (14). Finally, CCL11 is induced by IL-4 stimulation of equine respiratory epithelial cells and in P/i-stimulated equine Th cells (39), and its decrease may again point to a non-T2 endotype in EA. CCL11 downregulation in SEA was also identified in BALC macrophages by sc RNA sequencing (14). Nevertheless, the analysis of chemokine patterns in BALF alone does not directly allow for final conclusions on specific underlying mechanisms of EA due to the pleiotropic nature of chemokine sources. However, chemokines are highly potent immune mediators and provide potentially valuable and easily accessible markers in BALF, and the current findings warrant future characterisation of their cellular sources and targets.

The cellular analyses in the present study were limited by the available BALC from clinically well-characterised horses. Nevertheless, we identified T cell antigen reactivities for the first time together with Th polarisations in EA, which warrant T cell assessment in further cohorts for confirmation, particularly for the small group of MEA available here.

Typically, 2–5 × 10^7^ BALCs are recovered from one lavage with 200–300 ml saline, and 50–80% of the cryopreserved cells can be viably recovered after f-t. If all BALC (typically 2 × 10^7^ per horse after f-t) were used for stimulation *in vitro* with 2.5 × 10^6^ per stimulus (well), T cell responses to several antigens could be compared for most horses. For the present study, limited aliquots of BALC or PBMC were available for antigen restimulation experiments because other aliquots were used for different analyses. This prevented the comparison of more antigens. However, we prioritised the analysis of sufficient cell numbers per set-up over the comparison of more conditions considering the low frequencies of antigen-reactive T cells. In our opinion, this makes the interpretation of the results more reliable than the comparison of a higher number of antigens with fewer cells. Yet, this high cell number requirement limits the use of the assay developed here for larger screening panels.

The decreased CD4:CD8 ratio observed in SEA BALC compared with HE in the present study did not seem to limit the detection of antigen-reactive Th cell responses, as BALC CD4^+^cytokine^+^ net percentages were higher in SEA than HE. However, lower proportions of CD4^+^ T cells of BALC lymphocytes in SEA than in HE informed the calculation of CD4^+^cytokine^+^ cells as a percentage of all CD4^+^ T cells and not as a percentage of all lymphocytes. Although the CD4:CD8 ratio can be increased in human allergic asthma (47), it is not considered a major disease indicator in human BALC (48). To identify reasons for the decreased ratio in SEA BAL in the present study, the T cell subset viabilities were analysed separately. However, different viabilities were observed only in PBMC of MEA or SEA compared with HE, not in BALC, and do thus not explain the decreased CD4:CD8 ratio in SEA BALC. As the horses with SEA tended to be older than HE, age effects may have impacted their T cell populations. Inflamm-aging in horses over 20 years has been described and age is associated with increased CD4:CD8 ratios in equine PBMC (49). This is again in contrast to the present results of decreased CD4:CD8 ratios in the BALC from SEA, which were on average older than HE but still not considered elderly (49). In summary, the altered CD4:CD8 ratio in cryopreserved BALC lymphocytes observed here in SEA could not be elucidated but may be followed-up on to verify its importance, while this study focused on the functional cytokine expression capacities of T cells and antigen reactivity in EA.

### 4.1 Conclusions

The demonstration of *Aspergillus* antigen-reactive Th cell enrichment in the airways in SEA encourages a further more detailed evaluation of T cell antigens to obtain a more precise understanding of the etiology and pathogenesis of EA. The Th17 bias demonstrated here encourages further analysis of a Th17/neutrophil pathway in EA, including targeted therapeutic options. T cell characterisation further promotes the exploration of equine asthma as a model for neutrophilic T3 asthma in humans, which is otherwise difficult to model. To this end, further comprehensive identification of relevant T cell antigens would be beneficial and should be expanded. The development of an experimental approach to T cell analysis in this study can pave the way to a conclusive characterisation of T cell responses in EA to elucidate its pathogenesis and potential different endotypes or etiologies, including the different MEA and SEA phenotypes.

## Data Availability

The original contributions presented in the study are included in the article/**Supplementary Material**. Further inquiries can be directed to the corresponding author.
