# Supplementary material for: Aspergillus fumigatus antigen-reactive Th17 cells are enriched in bronchoalveolar lavage fluid in severe equine asthma
Source: Front Immunol. 2024 Aug 20;15:1367971. doi: 10.3389/fimmu.2024.1367971 (PMC11368783; doi:10.3389/fimmu.2024.1367971)
Supplement: Supplementary file 1 [file DataSheet1.pdf]

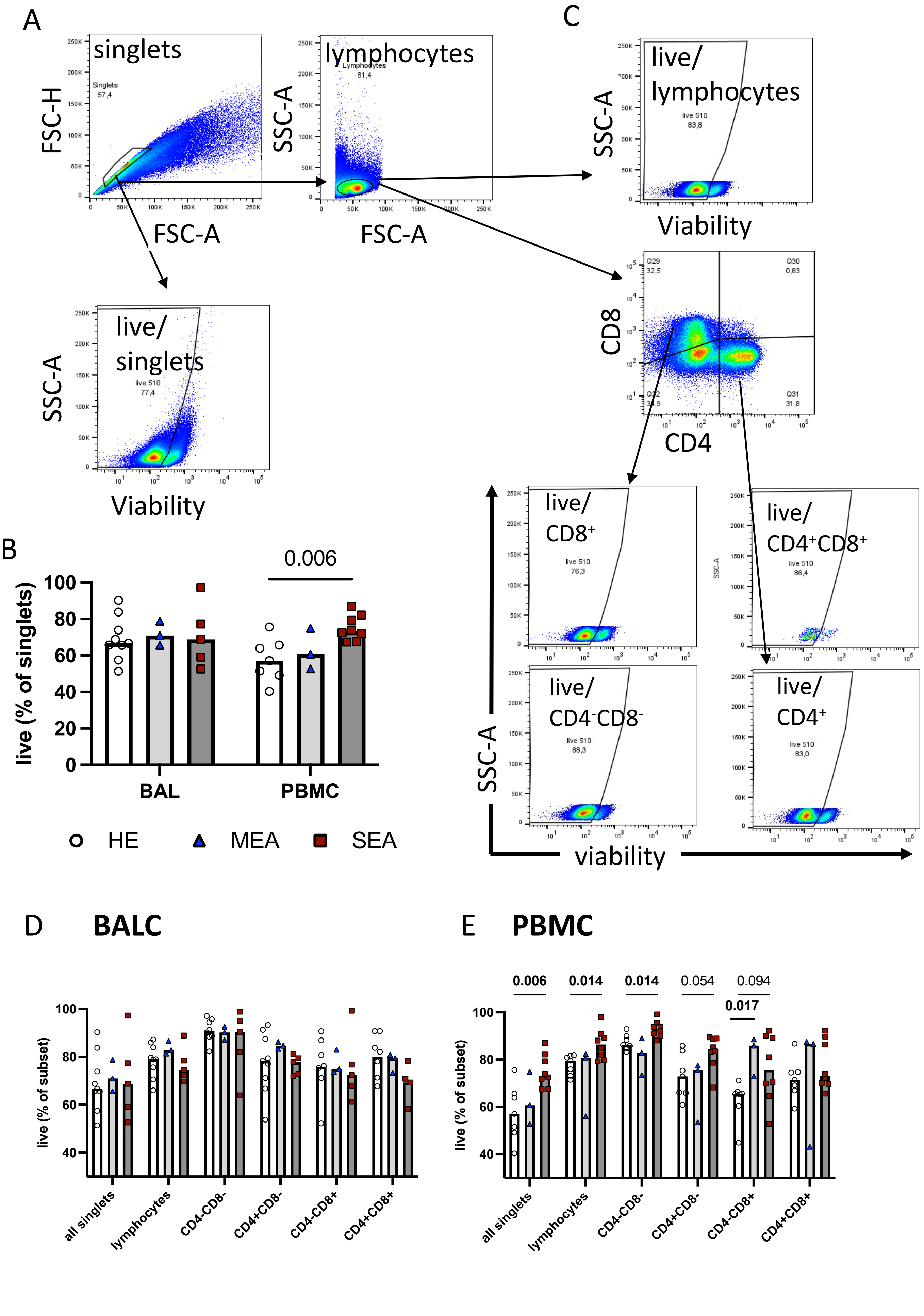

Supplementary Figure 1

Supplementary Figure 1 Cryopreserved equine PBMC lymphocytes from asthmatic horses have an increased viability Horses were characterized as healthy (HE, n=9), or to have mild-moderate equine asthma (MEA, n=3), or severe equine asthma (SEA, n=8). BALC and PBMC of the horses were cryopreserved, thawed, and subsequently analyzed by flow cytometry. A representative example of BALC (MEA) after freeze-thaw is shown. A) Singlets (conservative), and live cells were gated in a hierarchical manner for (B) the main viability analyses of all cells (compare Figure 1). (C) Alternatively, singlets, lymphocytes, CD4 vs. CD8 subsets, followed by live cell proportions were gated to evaluate each subset's viability in comparison. (D) Viabilities of BALC or (E) PBMC are plotted. The bars represent group medians, statistical comparisons in Mann-Whitney tests are indicated by lines with p-values (<0.1). The increased overall viability of SEA PBMC after thawing (B) was reflected in PBMC lymphocytes and particularly in $CD4^{-}CD8^{-}$ cells.

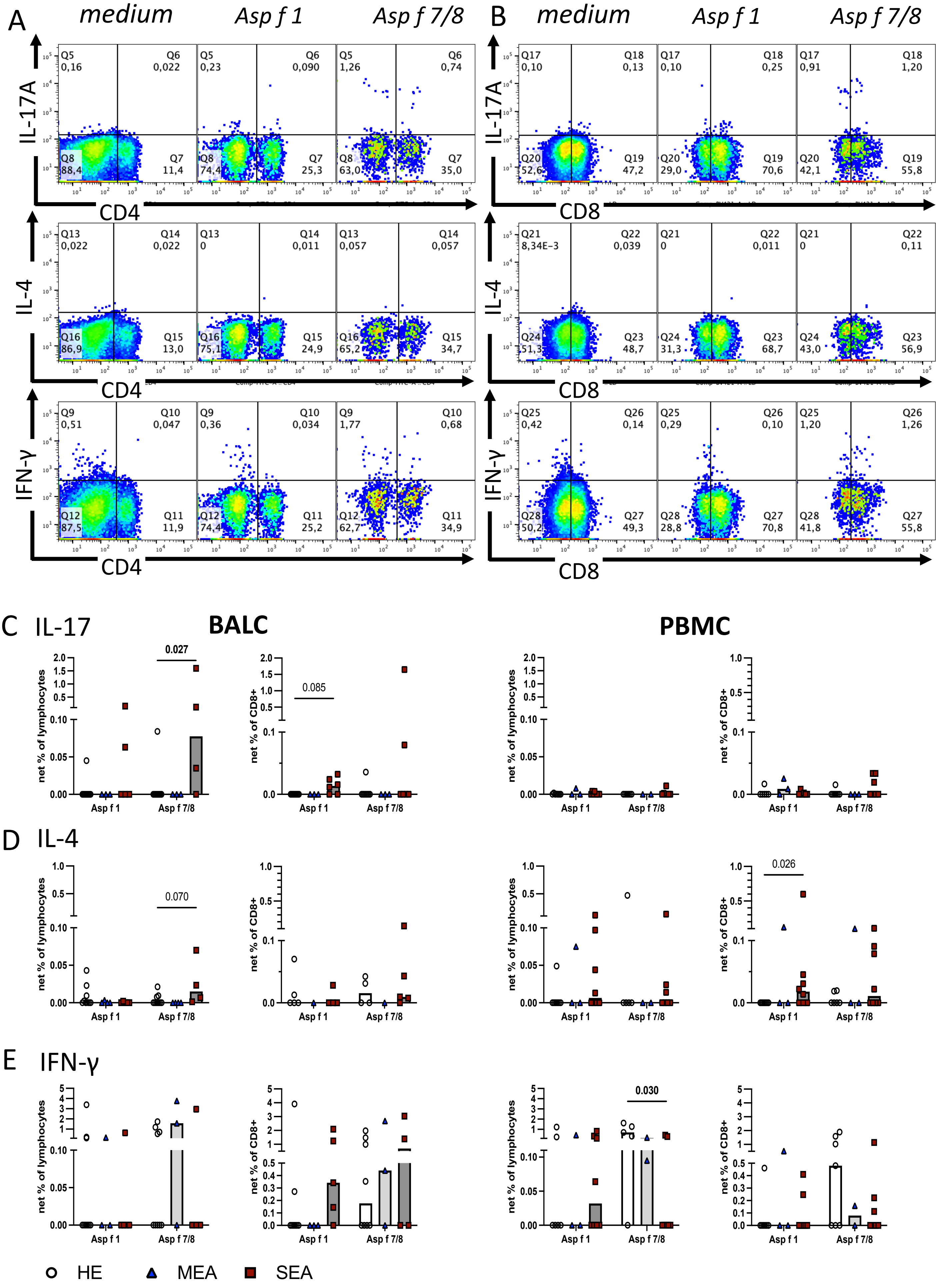

Supplementary Figure 2

Supplementary Figure 2 Lymphocytes express cytokines after antigen restimulation in equine BALC and PBMC Cryopreserved bronchoalveolar lavage cells (BALC), and PBMC from healthy horses (HE), or horses with mild-moderate (MEA), or severe equine asthma (SEA) were incubated *in vitro* with recombinant *Aspergillus fumigatus* antigens Asp f 1 or Asp f 7 mixed with Asp f 8 for 24 h, in comparison to medium alone and analyzed by flow cytometry. An example of BALC lymphocytes is shown (SEA, same as Figure 3 example, enlarged dots for visibility). Cytokines are plotted vs. (A) CD4, or (B) CD8. C–E) Stimulated cytokine expression was medium subtracted (net %) and analyzed in all live lymphocytes, and $CD8^+$ T cells. Bars represent group medians, statistical comparisons in Mann-Whitney-tests are indicated by lines with p-values if $p<0.1$.

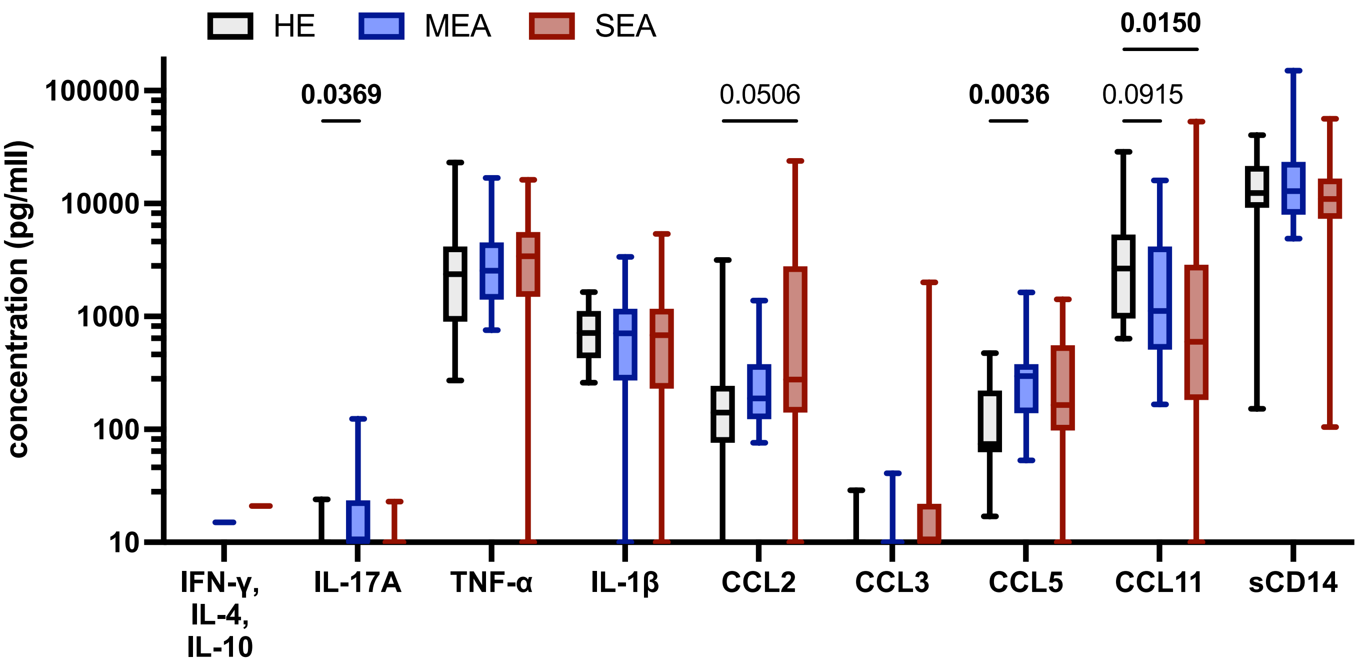

Supplementary Figure 3

Supplementary Figure 3 Soluble mediators in BALF from a larger cohort of horses confirm chemokine excess over cytokines

Soluble cytokines, chemokines, and sCD14 concentrations were quantified by bead-based assays in the BAL fluids (BALF) available from a larger number of horses (HE, n=19, MEA, n=20, SEA, n=23), including those presented in Figure 4. After log-transformation of the concentrations, group comparisons were performed by ANOVA with Fisher's LSD tests. IFN-γ, IL-4, and IL-10 were only detected in three samples, IL-17 was only detected in nine samples (1 HE, 6 MEA, 3 SEA), but was increased in MEA compared to HE. Chemokine concentrations were different between the groups: CCL2 tended to be increased in SEA, CCL5 was increased in MEA, and CCL11 was decreased in SEA compared to HE in agreement with the smaller subset of 20 samples with corresponding T cell analyses from the same horses (Figures 2, and 3).
